# Complete Genome Sequences of Two Genotype A2 Small Ruminant Lentiviruses Isolated from Infected U.S. Sheep

**DOI:** 10.1128/genomeA.00109-17

**Published:** 2017-03-30

**Authors:** Aspen M. Workman, Aaron M. Dickey, Michael P. Heaton, Michael L. Clawson, Timothy P. L. Smith

**Affiliations:** USDA, ARS, U.S. Meat Animal Research Center, Clay Center, Nebraska, USA

## Abstract

Two distinct subgroups of genotype A2 small ruminant lentiviruses (SRLVs) have been identified in the United States that infect sheep with specific host transmembrane protein 154 (*TMEM154*) diplotypes. Here, we report the first two complete genome sequences of SRLV strains infecting U.S. sheep belonging to genotype A2, subgroups 1 and 2.

## GENOME ANNOUNCEMENT

Small ruminant lentiviruses (SRLVs) infect sheep, goats, and wild ruminants, causing an incurable multisystemic chronic disease (1). SRLVs are phylogenetically divided into five groups, A to E, which are further divided into different subtypes based on their *gag* and *pol* sequences (2, 3). In the United States, genotypes A2 and B1 infect sheep and goats (4). Viral tropism and host susceptibility to the different SRLV strains are influenced by host and viral genotypes. In sheep, amino acid sequence variation encoded by the transmembrane protein 154 (*TMEM154*) gene is associated with susceptibility to SRLV A2 infection (5–9). On the pathogen side, two distinct SRLV A2 subgroups have evolved to infect sheep with specific *TMEM154* diplotypes (8). The lack of complete genome sequences for representatives of these viral subgroups has hampered progress in the identification of viral elements correlated with host adaptation associated with *TMEM154* diplotypes.

Viral genomic RNA was sequenced from the lungs of clinically ill sheep at the U.S. Meat Animal Research Center. These virus strains (USMARC-200303013-1 and USMARC-199906011-2, from subgroups 1 and 2, respectively) were part of the original study describing the association of SRLV genetic subgroups with *TMEM154* diplotypes (8). Encapsidated viral RNA was enriched in lung homogenates by nuclease treatment to degrade unprotected host and environmental nucleic acids. The remaining RNA was isolated using TRIzol LS (Life Technologies, Inc., Carlsbad, CA) and used to generate sequencing libraries according to the RNA Iso-Seq protocol (Pacific Biosciences, Menlo Park, CA), with one important modification. Fragment analysis revealed little RNA in the expected range of 9 kb for complete viral genomes. Therefore, poly(A) tails were added to the 3′ ends of the RNA using a Poly(A) polymerase tailing kit (Epicentre, Madison, WI) to allow cDNA synthesis of subgenomic fragments using the SMARTer PCR cDNA synthesis kit (Clontech, Mountain View, CA). The resulting cDNA was amplified then size fractionated with ELF (Sage Science, Beverly, MA). The largest fragments (approximately 2 to 3 kb) were used to generate SMRTbell libraries using the PacBio template prep kit. The SMRTbell templates were then sequenced using Pacific Biosciences RSII sequencing technology.

Reads were error-corrected using the SMRT Analysis server version 2.3.0 and imported into the Geneious software suite (version 9.1.7; Biomatters, Auckland, New Zealand). Adapter sequences and the added poly(A) tails were trimmed from reads using the BBDuk plugin (version 35.82) in Geneious. Reads greater than 1,000 nucleotides in length were assembled *de novo* with the Geneious assembler. All reads were then mapped to the *de novo* assembly, and the resulting consensus sequence was reported. The genome sizes of USMARC-200303013-1 and USMARC-199906011-2 were 9,206 and 9,185 nucleotides, with mean read lengths of 609 and 1,099 nucleotides, mapped read counts of 7,404 and 3,974, and mean read coverages of 617- and 721-fold, respectively. The complete genome sequences share 87% sequence identity and represent the first complete genome sequences for U.S. SRLV strains belonging to genotype A2.

### Accession number(s).

The sequences of USMARC-200303013-1 and USMARC-199906011-2 are deposited in GenBank/DDBJ/ENA under the accession numbers KY358787 and KY358788, respectively.
